# Septicemia due to *Aeromonas hydrophila* in a Pregnant Woman: A Case Report and Review of the Literature

**DOI:** 10.1155/S1064744995000743

**Published:** 1995

**Authors:** Ricardo Figueroa Damián, Roberto Villagrana Zesati, León Sáinchez Fernáindez, Jose Luis Arredondo García

**Affiliations:** Department of Infectious Diseases Instituto Nacional de Perinatologia Montes Urales No. 800 Col. Lomas de Virreyes, Delegación Miguel Hidalgo D.F. 11000 Mexico

## Abstract

*Background:* In the late 1960s, the first isolates of *Aeromonas* were recovered from human specimens. Presently, there is sufficient evidence to suggest that the different isolates of the genus *Aeromonas* are human pathogens. The most frequent site of infection is the digestive tract, although extraintestinal infection also occurs. In those cases involving septicemia, most infections occur in individuals with underlying diseases. This report presents the case of a pregnant woman with no underlying disease or signs of immunodeficiency who developed *A. hydrophila* septicemia at 24 weeks gestation.

*Case:* A 20-year-old pregnant woman was admitted with a history of 10 days of fever, chills, and diaphoresis. Three days before her hospitalization, she noted jaundice and choluria. Her liver was enlarged and her liver function tests were abnormal, with a moderate elevation of serum aminotransferases and direct serum bilirubin and a high serum alkaline phosphatase. Her blood and bone-marrow cultures revealed *A. hydropkila*. She was treated with parenteral ceftriaxone. She experienced a complete remission of her symptoms and laboratory abnormalities after therapy. The remainder of the pregnancy was normal. At 39.2 weeks gestation, she delivered a healthy male infant.

*Conclusion:* An association was noted between pregnancy and *A. hydrophila* septicemia in a woman without immunodeficiency or underlying disease, possibly indicating another infectious complication in pregnancy.


Infectious Diseases in Obstetrics and Gynecology 3:252–255 (1995)
© 1996 Wiley-Liss, Inc.

### Septicemia due to *Aeromonas hydrophila* in a Pregnant Woman: A Case Report and Review of the Literature

**Ricardo Figueroa Damián, Roberto Villagrana Zesati, León Sánchez Fernández, and Jose Luis Arredondo García**

*Department of Infectious Diseases, Instituto Nacional de Perinatologia, México, D.F., México*

ABSTRACT

*Background:* In the late 1960s, the first isolates of *Aeromonas* were recovered from human specimens. Presently, there is sufficient evidence to suggest that the different isolates of the genus *Aeromonas* are human pathogens. The most frequent site of infection is the digestive tract, although extra-intestinal infection also occurs. In those cases involving septicemia, most infections occur in individuals with underlying diseases. This report presents the case of a pregnant woman with no underlying disease or signs of immunodeficiency who developed *A. hydrophila* septicemia at 24 weeks gestation.

*Case:* A 20-year-old pregnant woman was admitted with a history of 10 days of fever, chills, and diaphoresis. Three days before her hospitalization, she noted jaundice and choluria. Her liver was enlarged and her liver function tests were abnormal, with a moderate elevation of serum aminotransferases and direct serum bilirubin and a high serum alkaline phosphatase. Her blood and bone-marrow cultures revealed *A. hydrophila*. She was treated with parenteral ceftriaxone. She experienced a complete remission of her symptoms and laboratory abnormalities after therapy. The remainder of the pregnancy was normal. At 39.2 weeks gestation, she delivered a healthy male infant.

*Conclusion:* An association was noted between pregnancy and *A. hydrophila* septicemia in a woman without immunodeficiency or underlying disease, possibly indicating another infectious complication in pregnancy. © 1996 Wiley-Liss, Inc.

KEY WORDS
Sepsis, immunodeficiency, jaundice

Members of the genus *Aeromonas* are gram-negative rods that belong to the family Vibrionaceae. They are oxidase-positive, facultative anaerobes with large zones of hemolysis around colonies on blood agar that ferment carbohydrates.[1,2]

*Aeromonas* was isolated in human feces for the first time in 1937. Not until 1980 was it recognized as a pathogenic bacteria causing gastrointestinal diseases in human beings.[1] The spectrum of intestinal diseases caused by *Aeromonas* ranges from acute, self-limited gastroenteritis of moderate intensity to chronic diarrhea that can persist for weeks or months.[3] With lesser frequency, aeromonads have been associated with extra-intestinal infections, resulting in peritonitis, osteomyelitis, skin and soft-tissue infections, meningitis, endocarditis, and septicemia.[1,4–6]

It has been suggested that patients who have immunosuppression or malignant diseases are more easily infected with aeromonads, although normal, healthy individuals may become infected as well. In cases of *Aeromonas* septicemia, many of the infec-

Address correspondence/reprint requests to Dr. Ricardo Figueroa Damián, Department of Infectious Diseases, Instituto Nacional de Perinatologia, Montes Urales No. 800, Col. Lomas de Virreyes, Delegación Miguel Hidalgo, C.P. 11000, México, D.F., México.

Received October 27, 1995
Accepted February 8, 1996

tions occur in individuals with neoplastic diseases,[7] while septicemia in patients without predisposing conditions is rare.[8]

A case of a pregnant woman without previous or concomitant disease, who developed an *A. hydrophila* septicemia at 24 weeks gestation is presented. A brief review of the literature on the association and incidence of *Aeromonas* infections and pregnancy is also presented.

### CASE REPORT

A 20-year-old primigravida at 24 weeks of gestation was admitted to the National Institute of Perinatology with a history of 10 days of fever, chills, diaphoresis, asthenia, and adynamia. Three days before her hospitalization, she noted choluria and jaundice and complained of a cough and pharyngeal pain. On her admission, she was agitated, and tachycardic with mental obtundation. She had dry oral mucosa, decreased skin turgor, and generalized jaundice. Her temperature was 38.5°C. Her examination revealed right upper-quandrant pain, an enlarged liver, and a pregnant uterus. A complete blood count revealed slight normochromic/normocytic anemia and a leukocyte count of 5,300/μl with 22% bands. Her liver function tests were abnormal, with a moderate elevation of serum aminotransferases (aspartate aminotransferase of 66 u/l and alanine aminotransferase of 52 u/l), an increase in direct serum bilirubin to 3.8 mg/dl, and a high serum alkaline phosphatase of 1,628 u/l. An abdominal ultrasound showed a normal biliary tract and an enlarged liver. A viable fetus with a gestational age of 18–20 weeks was observed. The discrepancy between the patient's reported gestational age and the ultrasound finding was attributable to her irregular menstrual cycle, making the true gestational age of the fetus uncertain.

The clinical picture was compatible with a sepsis syndrome. She was treated with parenteral ceftriaxone. On the 4th day of hospitalization, she became less febrile, and her blood and bone-marrow cultures revealed *A. hydrophila.* The bone-marrow culture was initially done to rule out typhoid fever. A stool culture was not done because the patient had no gastrointestinal symptoms. She experienced a complete remission of the signs and symptoms of sepsis and a normalization of her laboratory tests after therapy.

The serologic studies against hepatitis virus A, B, and C as well as anti-HIV were negative. The *A. hydrophila* isolated was resistant to gentamycin.

The remainder of her pregnancy was normal. At 39.2 weeks, she reentered the hospital and delivered a healthy male. The histopathologic study of the placenta was normal.

### DISCUSSION

In the last 2 decades, the genus *Aeromonas* has been increasingly recognized as a human pathogen. In the early 1980s, on the basis of biochemical characteristics, 4 phenotypes of *Aeromonas* were recognized: *A. hydrophila, A. sobria, A. caviae,* and *A. veronii.* Because of the marked differences in the polynucleotide sequences in each of these phenotypes, a new classification based on DNA-DNA reassociation kinetics was proposed: the hybridization group (HG).[9] For most institutions, the identification of *Aeromonas* isolates within the HG is impractical because of the technical time required and the costs of reagents and equipment. A feasible alternative is to identify aeromonads within the phenotypes.

Initially, aeromonads were identified as pathogenic in cold-blooded animals. In 1968, von Graevenitz and Mensch[10] reported the first series of *Aeromonas* isolates from human specimens. Of the 30 cases reported, 14 were of gastrointestinal origin. In 19 patients, the isolates were mixed with other bacteria. In 2 of them, both with hepatic cirrhosis, the isolation was from a blood culture.[10] Presently, there appears to be a consensus that aeromonads cause gastrointestinal disease. In addition to their pathogenic role, aeromonads have been found in the feces of so-called asymptomatic carriers.[3]

In 1988, the State of California established that an infection with *Aeromonas* was an obligatory reportable disease.[11] In California, the annual incidence of *Aeromonas* infection was 10.6 cases/million inhabitants. In 1 year, *Aeromonas* was isolated in the feces of 178 patients; in 19, from soft-tissue infections; in 11, from blood cultures; in 5, from bile; and in 12, from various other specimens including urine, sputum, eye, and placenta. Ninety percent of the patients with *Aeromonas* isolated from their feces also had gastrointestinal symptoms.[11]

After the digestive tract, the most frequent sites of aeromonad infections are the skin and soft tissues. The majority of these cases are associated with trauma in aquatic environments and contamination of injuries.[12–15] The spectrum of these infections include cellulitis, gas gangrene, myonecrosis, fulminant necrotizing infections, and osteomyelitis. Voss et al.[15] recently reported a series of 28 patients with musculoskeletal and soft-tissue *Aeromonas* infections, 23 (82%) of whom had acute open or penetrating injuries. In 13 of these, the initial trauma occurred in lake or river water. In the pathogenesis of this type of infection, it has been suggested that aeromonads produce hemolysins and cytotoxins that serve as virulent factors. These intracellular products may facilitate the tissue damage as well as muscle lesions.[16]

Septicemia due to *Aeromonas* is infrequent. Most cases are sporadic and community acquired, although nosocomial cases have been described.[4,7,8] *A. hydrophila* and *A. sobria* have been implicated as the species most frequently associated with septicemia.[8,17,18] *Aeromonas* septicemia is most likely to occur in patients who have immunocompromised or some underlying disease such as a malignancy, liver cirrhosis, or diabetes mellitus and perhaps in the elderly as a result of a decline in immune functions.[7,8,17,18]

Few cases of septicemia caused by *Aeromonas* have been described in patients without immunodeficiency. In the patient presented here, there was no underlying disease or history of immunodeficiency. In a series of 24 patients with *Aeromonas* bacteremia, only 1 was reported as not having a neoplastic disease.[7] In another study of 16 patients with septicemia, 8 had hematologic disorders and 6 had severe underlying illnesses, while only 2 were apparently healthy.[8] Thirteen patients with *Aeromonas* septicemia were reported from Australia in the years between 1983 and 1987. Chronic illnesses were present in 10 of these. Of the 3 remaining patients, 2 presented with cholangitis and the third, an otherwise healthy 20-year-old male, developed fever 4 days after an umcomplicated tonsilectomy.[18]

In all cases reported in the literature, the mortality was significanly associated with the severity of the underlying disease. In the epidemiologic studies of *Aeromonas* infection reported from the State of California, the mortality rate was 2.3%, with 5 deaths occurring in hospitalized patients with severe medical disorders.[11] Three of the deaths occurred among 11 patients who developed septicemia, giving a mortality rate for this group of 27.3%.[11] The mortality rate with *Aeromonas* septicemia reported in other series of cases ranged from 27 to 56%.[7,8]

In some cases of *Aeromonas* septicemia, the origin of the infection is evident, e.g., from an abscess, cellulitis, infected ulcer, or empyema. A minority of cases developed septicemia or skin lesions compatible with ecthyma gangrenosum.[7,8,18] In the case reported here, the principal manifestation was jaundice, an infrequent sign in this type of infection. Harris et al.,[7] in their study of 24 patients, identified only 1 patient with jaundice. In the series reported by Vilasecal-Momplet et al.,[8] only 2 of 16 patients presented with jaundice secondary to cholangitis. In these patients, an infection of the intrahepatic biliary tract was the origin of the septicemia. The moderate alterations in the liver function tests presented by our patient and the correction of these laboratory abnormalities after therapy suggest that the liver function abnormalities resulted from cholestasis secondary to sepsis.

In a review of the medical literature using MEDLINE CD-ROMM and *Index Medicus* from 1970 to 1994, we found no report of an association between *Aeromonas* septicemia and pregnancy. However, there were reports of the isolation of *Aeromonas* in septic abortion material[8] and placenta.[11] In an abstract of an infectious diseases conference just 1 case was reported of a pregnant woman who died as a result of a septic abortion in whom *A. hydrophila* was isolated from a blood culture.[19]

The vulnerability of pregnant women to infections depends on many host factors, including past and present immunologic status, hormonal and hematologic changes occurring during pregnancy, and perhaps dietary habits. Some altered immune responses during pregnancy involve a decrease in specific antibody titers, a decrease in total T-lymphocytes, and a decrease in helper-suppressor ratios, as well as a decrease in the chemotactic and phagocytic properties of granulocytes.[20]

In the case reported here, the origin of the sepsis is still unknown. In the majority of the septicemia cases we reviewed, the origin was considered to be endogenous, principally from the patient's digestive tract.

*Aeromonas* species are usually susceptible in vitro to third-generation cephalosporins, quinolones, and aminoglycosides, as well to the new β-lactam antibiotics such as aztreonam and imipenem.[21]

### REFERENCES

1. McGowan J, Steinberg J: Other gram-negative bacilli. In Mandell G, Bennett JE, Dolin R (eds): Principles and Practice of the Infectious Diseases. 4th ed. New York: Churchill Livingstone, pp 2106–2117, 1995.
2. Janda JM, Abbott S, Carnahan A: *Aeromonas* and *Plesiomonas*. In Murray P, Baron EJ, Pfaller M, Tenover F, Yolken RN (eds): Manual of Clinical Microbiology. 6th ed. Washington, DC: American Society for Microbiology, pp 477–482, 1995.
3. Moyer N: Clinical significance of *Aeromonas* species isolated from patients with diarrhea. J Clin Microbiol 25:2044–2048, 1987.
4. Baddour L: Extraintestinal aeromonal infections—Looking for Mr. Sandbar. Mayo Clin Proc 67:496–497, 1992.
5. Muñoz P, Fernández-Baca V, Pelaez T, Sánchez R, Rodríguez-Creixems M, Bouza E: *Aeromonas* peritonitis. Clin Infect Dis 18;32–37, 1994.
6. Parras F, Díaz MD, Reina J, Moreno S, Guerrero C, Bouza E: Meningitis due to *Aeromonas* species: Case report and review. Clin Infect Dis 17:1058–1060, 1993.
7. Harris R, Fainstein V, Elting L, Hopfer R, Bodey G: Bacteremia caused by *Aeromonas* species in hospitalized cancer patients. Rev Infect Dis 7:314–320, 1985.
8. Vilaseca-Momplet J, Arnau de Bolos JM, Fernández-Pérez F, Andreu-Domingo A, López-Vivancos J, López-Hernández A: Bacteremia por *Aeromonas hydrophila*. Características clínicas y bacteriológicas. A propósito de 16 observaciones. Rev Clin Esp 177:104–107, 1985.
9. Janda JM: Recent advances in the study of the taxonomy, pathogenicity and infectious syndromes associated with the genus *Aeromonas*. Clin Microbiol Rev 4:397–410, 1991.
10. Von Graevenitz A, Mensch A: The genus *Aeromonas* in human bacteriology. N Engl J Med 278:245–249, 1968.
11. King G, Werner B, Kizer K: Epidemiology of *Aeromonas* infections in California. Clin Infect Dis 15:449–452, 1992.
12. Cailleaux V, Dupont MJ, Hory B, Amsallem D, Michel-Briand Y: Why did infection with *Aeromonas hydrophila* occur when water contains so many other microorganisms? Clin Infect Dis 16:174, 1993.
13. Newton JA, Kennedy Ch: Wound infection due to *Aeromonas sobria*. Clin Infect Dis 17:1082–1083, 1993.
14. Fraire AE: *Aeromonas hydrophila* infection. JAMA 239:192, 1978.
15. Voss L, Rhodes KH, Johnson A: Musculoskeletal and soft tissue *Aeromonas* infection: An environmental disease. Mayo Clin Proc 67:422–427, 1992.
16. Gold WL, Salit IE: *Aeromonas hydrophila* infections of skin and soft tissue: Report of 11 cases and review. Clin Infect Dis 16:69–73, 1993.
17. Ketover BP, Young LS, Armstrong D: Septicemia due to *Aeromonas hydrophila:* Clinical and immunologic aspects. J Infect Dis 127:284–289, 1973.
18. Dryden M, Munro R: *Aeromonas* septicemia: Relationship of species and clinical features. Pathology 21:111–114, 1989.
19. Chang J, Gomar G, Keller CA, Ramirez CA: Fatal sepsis due to *Aeromonas hydrophila* in nonimmunosuppressed patient. In: Program and Abstracts of the 26th Interscience Conference on Antimicrobial Agents and Chemotherapy. Washington, DC: American Society for Microbiology, 1986.
20. Brabin G: Epidemiology of infection in pregnancy. Rev Infect Dis 7:579–603, 1985.
21. Brugos A, Quindos G, Martinez R, Rojo P, Cisterna R: In vitro susceptibility of *Aeromonas caviae, Aeromonas hydrophila* and *Aeromonas sobria* to fifteen antibacterial agents. Eur J Clin Microbiol Infect Dis 9:413–416, 1990.
